# Embryonic desiccation resistance in *Aedes aegypti*: presumptive role of the chitinized Serosal Cuticle

**DOI:** 10.1186/1471-213X-8-82

**Published:** 2008-09-13

**Authors:** Gustavo Lazzaro Rezende, Ademir Jesus Martins, Carla Gentile, Luana Cristina Farnesi, Marcelo Pelajo-Machado, Alexandre Afrânio Peixoto, Denise Valle

**Affiliations:** 1 Laboratório de Fisiologia e Controle de Artrópodes Vetores, Instituto Oswaldo Cruz, FIOCRUZ and Laboratório de Entomologia, Instituto de Biologia do Exército, Rio de Janeiro, Brazil; 2 Laboratório de Biologia Molecular de Insetos, Instituto Oswaldo Cruz, FIOCRUZ, Rio de Janeiro, Brazil; 3 Laboratório de Patologia, Instituto Oswaldo Cruz, FIOCRUZ, Rio de Janeiro, Brazil; 4 School of Biological and Chemical Sciences, Queen Mary University, 327 Mile End Road, London E1 4NS

## Abstract

**Background:**

One of the major problems concerning dengue transmission is that embryos of its main vector, the mosquito *Aedes aegypti*, resist desiccation, surviving several months under dry conditions. The serosal cuticle (SC) contributes to mosquito egg desiccation resistance, but the kinetics of SC secretion during embryogenesis is unknown. It has been argued that mosquito SC contains chitin as one of its components, however conclusive evidence is still missing.

**Results:**

We observed an abrupt acquisition of desiccation resistance during *Ae. aegypti *embryogenesis associated with serosal cuticle secretion, occurring at complete germ band extension, between 11 and 13 hours after egglaying. After SC formation embryos are viable on dry for at least several days. The presence of chitin as one of the SC constituents was confirmed through Calcofluor and WGA labeling and chitin quantitation. The *Ae. aegypti *Chitin Synthase A gene (*AaCHS1*) possesses two alternatively spliced variants, *AaCHS1a *and *AaCHS1b*, differentially expressed during *Ae. aegypti *embryonic development. It was verified that at the moment of serosal cuticle formation, *AaCHS1a *is the sole variant specifically expressed.

**Conclusion:**

In addition to the peritrophic matrix and exoskeleton, these findings confirm chitin is also present in the mosquito serosal cuticle. They also point to the role of the chitinized SC in the desiccation resistance of *Ae. aegypti *eggs. *AaCHS1a *expression would be responsible for SC chitin synthesis. With this embryological approach we expect to shed new light regarding this important physiological process related to the *Ae. aegypti *life cycle.

## Background

The mosquito *Aedes aegypti *is the main dengue vector. One of the major problems concerning dengue transmission is that *Ae. aegypti *eggs resist desiccation, surviving several months under dry conditions in a dormancy state at the end of their embryonic development [1-3]. An important component of the desiccation resistance is the serosal cuticle (SC), a layer covering the embryo that is synthesized at early embryogenesis [4-7] (for review see [2]). The serosal cuticle is secreted by the serosa, a membrane formed by extra-embryonic cells that surrounds the whole embryo of many insects [2,8-11]. Upon oviposition, the mosquito eggshell is composed of two layers, a compound exochorion and a smooth endochorion [12]. The SC develops during embryogenesis and becomes the third eggshell layer, lying beneath the endochorion [2,4]. It should be noted that the terminology of mosquito eggshell coverings, including the serosa and SC, is a matter of literary controversy. For example, Telford [7] designates the serosal cuticle as "vitelline membrane", Beckel [4] refers to the serosal cuticle as "transparent cuticle" while Harwood [13] and Harwood and Horsfall [5] name the endochorion and serosal cuticle as "chorion" and "endochorion", respectively. We follow the revised nomenclature, described by Clements [2] and Monnerat et al [14]. It is also important to emphasize that the serosal cuticle is not considered an "embryonic cuticle" *per se*, since it is secreted by the extraembryonic serosa cells and not by the embryo itself, as true embryonic cuticles [15].

Mosquito egg desiccation resistance is attributed to the serosal cuticle, which impedes water to escape from the embryo [3-5,7,16]. However, it was recently claimed that 2-hour old embryos or even oocytes are impermeable to water due to their covering layers [17,18].

Unlike the chorion, the SC is resistant to chlorine digestion, this being precisely the property employed to identify it [4-6]. Although it was argued in the 1950's that mosquito SC contains chitin as one of its components [4,13], conclusive evidence is still missing. Moreover, the kinetics of SC secretion during embryogenesis is unknown.

Chitin is a homo-amino-polysaccharide formed by β-1,4 linked units of N-acetyl-D-glucosamine (GlcNAc) [19]. It is the second most abundant biopolymer in nature, present in fungi, nematodes and arthropods [20-22]. Chitin Synthase (CHS) is the enzyme responsible for the chitin polymer formation, using UDP-GlcNAc as sugar donors [19,23]. In insects chitin is present in the exoskeleton as well as in the peritrophic matrix (in the midgut), and it is synthesized, respectively, by one of the two classes of Chitin Synthase genes, CHS-A and CHS-B [23-28]. Insect CHS-A genes have two mutually exclusive exons, resulting in two mRNA spliced variants. Both exons code for 59 amino acids comprising an extracellular, a transmembrane and an intracellular domain located near the carboxy terminus of the protein. The two spliced variants are expressed throughout *Tribolium castaneum *and *Manduca sexta *development [24,26]. The deletion of the *Drosophila melanogaster *CHS-A gene, named *kkv*, is embryonic lethal [29,30] indicating a crucial role of this gene in development.

In opposition to the model system *D. melanogaster*, little is known about molecular mechanisms that control mosquito embryogenesis. Although morphological movements are documented (for review see [2]), papers describing some of the molecules associated with embryogenesis in mosquitoes are very recent [31-34]. Lack of molecular studies with mosquito embryos is mainly due to the hard and impermeable nature of their eggshell, a characteristic that hampers fixation [14] and internalization of probes. Both procedures are necessary for *in situ *hybridization or immunostaining protocols, adopted for the investigation of gene expression profiles.

In summary, the eggshell of mosquitoes is a physical barrier for embryological studies, and the serosal cuticle, which is one of the eggshell constituents, is supposed to be important in *Ae. aegypti *egg resistance to desiccation. We thus opted to better understand SC nature, physiological role and developmental kinetics in order to investigate its properties and relevance during mosquito embryonic development.

## Results

### Acquisition of desiccation resistance is associated to serosal cuticle formation

**Figure 1:**
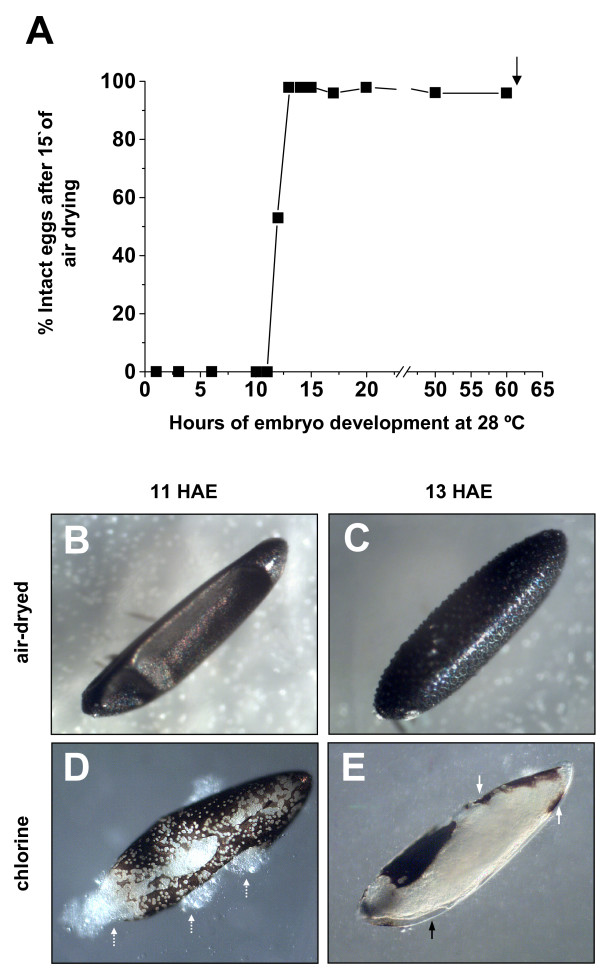
**Abrupt acquisition of desiccation resistance in *Aedes aegypti *embryogenesis is related to serosal cuticle formation**. Pools of synchronized eggs at different embryonic ages were air-dried for 15 minutes (see items 2 and 3 of Methods). (**A**) The percentage of intact eggs was evaluated. Black arrow: end of embryogenesis. (**B**) 11-HAE and (**C**) 13-HAE eggs after 15 minutes air-exposure. The abrupt acquisition of impermeability between 11 and 13 hours of development is coincident with the appearance of the serosal cuticle, as determined by chlorine digestion (item 5 of Methods). (**D**) 11-HAE egg after 15 minutes exposure to chlorine. White dashed arrows: extrusion of cellular contents. (**E**) 13-HAE egg exposed to chlorine for 30 minutes. Note chorion almost complete disintegration. Black arrow: transparent serosal cuticle around the embryo. White arrows: reminiscent chorion.

Quantification of desiccation resistance, performed through air exposure of differently aged eggs (Fig. 1, see items 2 and 3 of Methods for details), resulted in complete shrinkage of eggs up to 11 HAE (HAE = hours after egglaying) (Fig. 1A, B). In contrast virtually all the eggs 13 HAE or older remained intact (Fig. 1A, C). It should be note that in our conditions *Ae. aegypti *total embryonic development takes 61.5 hours at 28°C (Farnesi and Rezende, to be published elsewhere).

The serosal cuticle, but not mosquito chorion, is resistant to chlorine digestion [4,6,13], and this approach was used to further evaluate 11 and 13 HAE eggs (item 5 of Methods). Eleven HAE eggs left in the chlorine solution (bleach) for 10–20 minutes start to disintegrate (Fig. 1D) and if left for a longer period (30 minutes) disintegrate completely, both eggshell and embryo (data not shown). In contrast a 13 HAE embryo exposed to bleach for 30 minutes remains intact and is clearly visible beneath the transparent serosal cuticle, after almost complete digestion of the chorion (Fig. 1E). Taken together, these results indicate the SC is associated to desiccation resistance acquisition that abruptly appears between 11 and 13 hours after egglaying.

**Figure 2:**
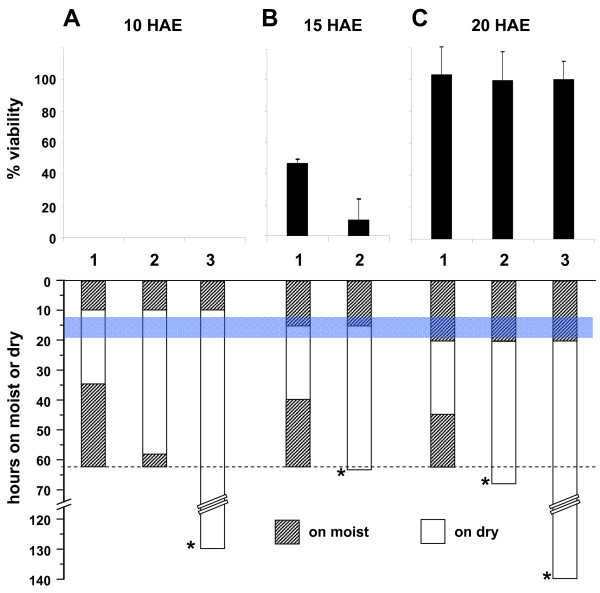
**Embryo viability in dry conditions is greatly improved after serosal cuticle formation**. Pools of synchronized developing eggs were transferred to dry conditions after 10, 15 or 20 HAE (A, B, C, respectively), a period that encompasses SC formation. In each case, embryos were kept on dry for 25 (1), 48 (2) or 120 (3) hours, as indicated. Hatching of samples was then checked (see item 4 of Methods for details). Data are expressed as percent viability, corrected from control samples, kept on moist conditions throughout development. Blue stripe: putative period between beginning of SC formation and its complete maturation (see Discussion). Dashed line: end of embryogenesis. Hatching stimuli was performed at the end of embryogenesis, or when indicated by *.

To evaluate the physiological relevance of desiccation resistance acquisition, we quantified the viability of developing eggs transferred to dry conditions at different ages and for varying periods of time, from 25 to 120 hours (Fig. 2, see item 4 of Methods for details). No hatching was observed if eggs were exposed to dry conditions before SC formation (10 HAE, Fig 2A). In contrast, samples dried at 20 HAE were fully viable (Fig 2C). Intermediate values were obtained if eggs were transferred to dry conditions in a period close to SC formation (15 HAE, Fig 2B). In this case viability was inversely proportional to the period of time spent in dry conditions. Since 120 hours on dry, starting from 20 HAE, corresponds to approximately 3 days after the end of embryogenesis, we conclude that the fully formed SC at 20 HAE is competent to impede embryonic and larvae desiccation during and after completion of embryogenesis (see Discussion).

### The serosal cuticle is formed at the moment of complete germ band extension

**Figure 3:**
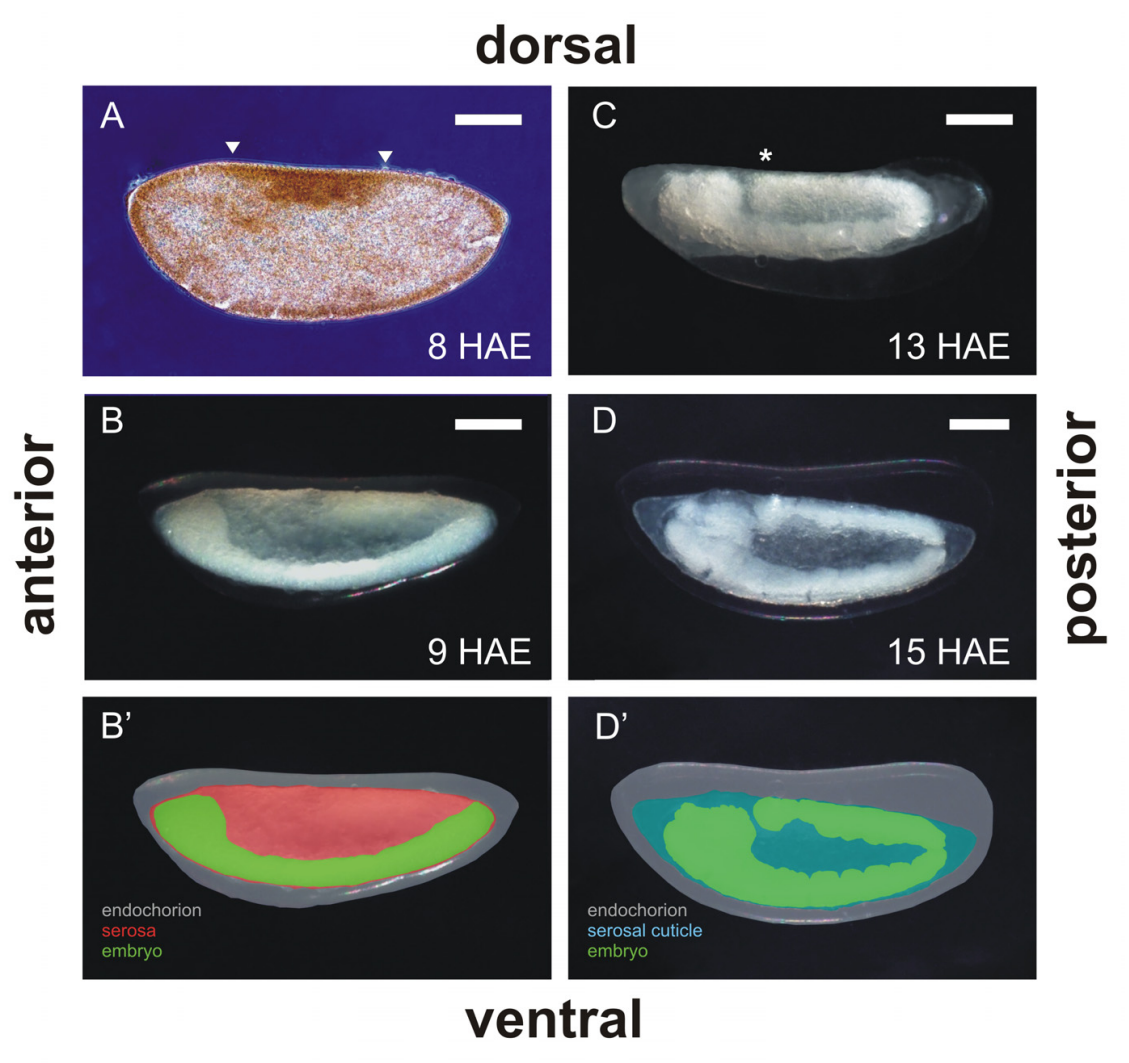
**Serosal cuticle is formed at complete germ band extension**. Clarified eggs at different embryonic ages (item 6 of Methods): (**A**) 8-HAE embryo, showing the serosa anlage (arrowheads). (**B, B'**) 9-HAE embryo at germ band extension, showing complete formation of serosa. (**C**) 13-HAE embryo at complete germ band extension. *: note posterior tip of germ band adjacent to the embryonic head. (**D, D'**) 15-HAE embryo at the beginning of germ band retraction. In B' and D', colors aim to better visualize embryo surrounding layers. In D', the serosa is located bellow the serosal cuticle. Bar = 100 μm

**Figure 4:**
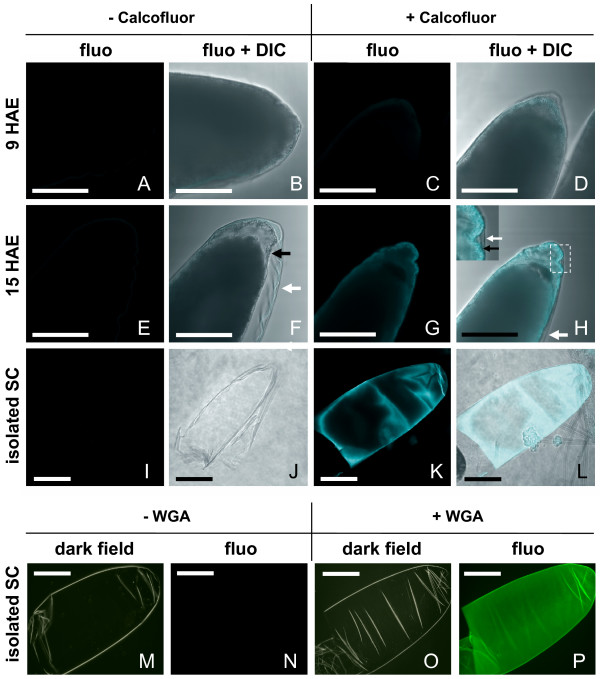
**Chitin is present in serosal cuticle**. (A-L) Calcofluor was used to reveal chitin in the serosal cuticle: labeling of embryos (A-H) or isolated SC (I-L). Panels exhibit fluorescence (fluo) alone or together with DIC (fluo + DIC), as indicated. Negative controls (-Calcofluor) confirm absence of auto-fluorescence in all samples. (**A-D**) Posterior half of clarified 9-HAE; (**E-H**) 15-HAE embryos; (**I-L**) Isolated SC from embryos at the end of development, broken at the line of dehiscence, without the egg shell cap. **J **is a lateral view. (M-P) WGA staining of isolated SC. Panels exhibit dark field or fluorescence (fluo), as indicated. (**M, N**) Non labeled control; (**O, P**) Labeled SC. See item 7 of Methods. Black arrow: serosal cuticle. White arrow: clarified endochorion. Bar = 100 μm.

In order to determine the embryonic development stage related to SC formation, embryos were clarified as detailed in Methods, item 6 (Figure 3). Embryos at 8 HAE exhibited the beginning of a morphological differentiation of the serosa anlage, immediately after the cellular blastoderm stage (Fig. 3A). This serosa anlage was also deduced in *An. gambiae *embryos from the expression of serosa specific genes [32,33] (see also Discussion). Subsequently (9-HAE embryos) germ band extension starts, and the serosa appears fully formed, completely surrounding the embryo (Fig. 3B, B'). At 13 HAE, the germ band extension is complete, as noted by the adjacent positions of the embryo head and its posterior end (at the dorsal embryo side), and the serosal cuticle is being formed (Fig. 3C). At 15 HAE the serosal cuticle is completely formed (Fig. 3D, see also Figures 1 and 4). The scheme in D' was deduced from the results shown in Figure 1 and 4 (see below).

### Chitin is present in the serosal cuticle

Different methods were used to confirm the presence of chitin in mosquito SC, as previously stated by Beckel [4] and Harwood [13], see item 7 of Methods. The first approach consisted of labeling with Calcofluor White, a fluorescent molecule that binds β-1,4-linked D-glycopyranoside units [35]. Since some mosquito embryonic structures have autofluorescence [36], controls were elaborated to confirm specific Calcofluor labeling. Figure 4 compares fluorescence of embryos before or after incubation with Calcofluor. In both cases fluorescence images are shown either alone or together with DIC microscopy. Clarified 9-HAE embryos incubated with Calcofluor display a very faint fluorescence in serosal cells (Fig. 4A–D) whereas 15-HAE embryos in the same conditions exhibit specific fluorescence in the SC (Figs. 4E–H). Moreover, isolated serosal cuticle derived from fully developed embryos exhibit intense fluorescence (Fig. 4I–L). Isolated SCs were also submitted to staining with WGA, a highly specific lectin for N-acetyl-D-glucosamine polymers [37,38], showing uniform labeling (Fig. 4M–P).

Finally, quantitation of chitin content of isolated SCs was performed according to Lehmann and White [39]. With this approach we detected 3.24 (± 0.08) ng of chitin, as corresponding glucosamine, for each isolated SC.

### Sequence and organization of the *AaCHS1 *gene

There are two *CHS *genes in the *Ae. aegypti *genome [40], *AaCHS1 *and *AaCHS2 *(see item 8 of Methods for details of nomenclature). The latter, a Class B CHS gene, is associated with chitin production in the midgut [41]. Specific primers could not detect expression of *AaCHS2 *during *Ae. aegypti *embryogenesis (see Additional File 1, Figure 1)

**Figure 5:**
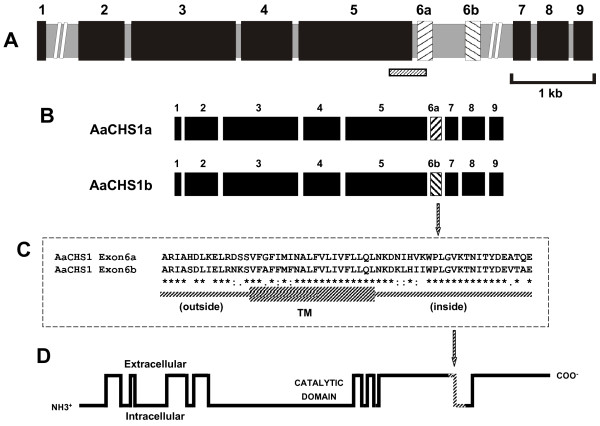
**Deduced gene structure, alternative splice and putative transmembrane protein profile of *AaCHS1***. (**A**) Gene structure: black boxes indicate constitutive exons, hatched boxes indicate mutually exclusive exons and gray boxes indicate introns. *AaCHS1 *is composed of ten exons (102; 534; 1,215; 594; 1,320; 177; 177; 204; 363 and 222 bp) and nine introns (28,587; 79; 61; 73; 58; 378; 5,620; 75 and 57 bp). Introns 1 and 7 are out of scale. The hatched bar underneath exons 5 and 6a marks the location of the fragment amplified by degenerate PCR. (**B**) Two options for *AaCHS1 *alternatively spliced edition: in *AaCHS1a e*xon 5 is spliced with exon 6a and 7, leaving exon 6b outside while in *AaCHS1b e*xon 5 is spliced with exon 6b and 7. (**C**) Alignment of predicted amino acid sequence of AaCHS1 exons 6a and 6b (see Additional File 2, Figure 2) using ClustalW software. TM: transmembrane alpha-helix span. Symbols below the aligned amino acid sequences indicate identical (*), highly conserved (:) and conserved (.) residues. (**D**) Predicted profile of AeCHS1 protein: transmembrane helices are indicated as vertical bars and regions inside or outside of the plasma membrane by horizontal bars. The hatched portion of the protein indicates the region coded by exon 6a or 6b (a segment containing a transmembrane domain).

Before the release of the *Ae. aegypti *genome [40], degenerated primers designed to amplify a conserved region of Dipteran CHS-A genes were applied on *Ae. aegypti *genomic DNA to look for a putative *AaCHS1 *gene. An *AaCHS1 *fragment was obtained and employed to design specific primers that detected the presence of both *AaCHS1a *and *AaCHS1b *mRNA in embryonic development (see Additional File 1, Figure 1). Afterwards, using *Drosophila melanogaster kkv *(*Dm_kkv*) sequence as the subject, the entire putative *AaCHS1 *was found in the *Ae. aegypti *genome project. Its deduced amino acid sequence was compared with *An. gambiae *CHS-A (*AgCHS1*) and *Dm_kkv*, demonstrating a high degree of similarity among dipterans (see Additional file 2, Figure 2). The schematic representation of the genomic structure of *AaCHS1 *is shown in Fig. 5A. The first intron contains an open reading frame similar to the pol-like *MosquI-Aa2 *protein, a non-LTR retrotransposon element [42] (data not shown).

Details of the *AaCHS1 *mutually exclusive exon 6a/6b organization, sequence and deduced transmembrane domains are displayed in Fig. 5B–D. The AaCHS1 protein can be subdivided into three domains: an N-terminal region containing eight transmembrane domains, a central cytosolic region with the catalytic domain, characterized by the QRRRW chitin synthase signature motif [19], and a C-terminal region containing seven additional transmembrane domains. Five transmembrane domains of the C-terminal region are clustered together close to the catalytic domain whereas the two other transmembrane domains are located 217 amino acids downstream. Both exons, 6a and 6b, translate 59 amino acids that encompass the penultimate transmembrane domain of the protein (Fig. 5C, D). The amino acid sequences encoded by exons 6a and 6b have 78% identity (Fig. 5C).

### *AaCHS1 *mRNA expression during embryonic development

**Figure 6:**
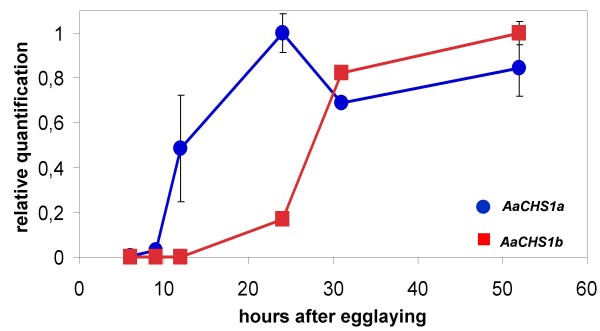
**Only the *AaCHS1a *splice variant is expressed when serosa is formed**. The relative quantitation of *AaCHS1a *and *AaCHS1b *expression was analyzed by qPCR. *AaCHS1a *has a slight expression at 6-HAE. At 9-HAE on its levels increase significantly, suggesting an *AaCHS1a *role in synthesis of chitin to the Serosal Cuticle. *AaCHS1b *is absent until 12-HAE. Both isoforms are expressed in late embryogenesis (31 and 52 hours). Bars represent the mean relative abundance +/- the range based on the SEM (Standard Error of the Mean).

Messenger RNA abundance in differently aged eggs was analyzed through qPCR with primers for *AaCHS1a *or *AaCHS1b*. Embryos belonging to three groups, related both to the kinetics of desiccation resistance acquisition and SC formation, were evaluated before (6 and 9 HAE), during (12 HAE) and after (24, 31 and 52 HAE) the abrupt desiccation resistance acquisition, exhibited in Figure 1A. The *AaCHS1 *alternative spliced forms are differentially expressed during *Ae. aegypti *embryogenesis (Fig. 6). A slight expression of *AaCHS1a *is noted at 9 HAE, increasing significantly up to 24 HAE, a period that encompasses the complete serosa formation, at 9 HAE (Fig. 3B), and the abrupt desiccation resistance acquisition (11–13 HAE, Fig. 1A). Thus, *AaCHS1a *expression attains its higher rate of increase after the serosa has completely surrounded the embryo. This corresponds to the moment of serosal cuticle synthesis (see Fig. 4C and 4G). In contrast, there is no *AaCHS1b *expression up to 12 HAE, and its mRNA is only slightly expressed at 24 HAE, both isoforms being expressed at late embryogenesis, at 31 and 52 HAE, respectively at the moments of dorsal closure and embryo organogenesis (see Additional File 3, Figure 3).

## Discussion

### Chitin synthesis in *Ae. aegypti *embryogenesis

Beckel [4] and Harwood [13] have previously pointed to the presence of chitin in *Aedes *SC. We have conclusively confirmed this issue through Calcofluor and WGA labeling and direct chitin quantitation. In a recent paper Moreira et al conclude that a chitin-like material is present in eggshells right from oviposition [18], possibly being part of the compound exochorion or the smooth endochorion [12,14]. As stated by Moreira et al, their data "do not rule out a subsequent synthesis of chitin by serosa" [18] which, according to the data presented here seems to be the case.

A fragment of the *AaCHS1 *genomic DNA was sequenced, and the whole putative *AaCHS1 *gene (class CHS-A) was subsequently obtained from *Ae. aegypti *genome according to alignment of predicted amino acids with dipteran CHS-A genes. CHS-A orthologues from *T. castaneum *and *M. sexta *possess two splice variants [24-26], these same variants being expressed during *Ae. aegypti *embryogenesis. The difference between both is a region of 59 amino acids codified by different exons in each alternative mRNA.

There is no experimental evidence regarding the specific use of any of the two transcripts. However, specific RNAi for each of the two *T. castaneum *transcripts, *TcCHS1a *and *TcCHS1b*, display distinct phenotypes when dsRNA specific for each transcript are injected into larval and pupal stages [25]. *TcCHS1b *is required only during pupal-adult molt where its absence leads to death at this stage; *TcCHS1a *is required both for larval-pupal and pupal-adult moults, its lack leading to death at both stages, indicating distinct roles for each transcript [25]. Moreover, during both *T. castaneum *and *M. sexta *development the transcript containing the first alternative exon is far more expressed than the mRNA with the second one [24-26], a trend that was also observed during *Ae. aegypti *embryogenesis, see below.

Similar to the *T. castaneum TcCHS2 *gene [24], synthesis of *Ae. aegypti *CHS-B, *AaCHS2 *[41], was not found during embryogenesis. Accordingly, RNAi experiments with *T. castaneum *larvae demonstrated that the CHS-B *TcCHS2 *gene is required only for peritrophic matrix chitin synthesis [25].

The high increase of *AaCHS1*a expression at 9–12 HAE is concomitant with the appearance of the chitinized SC (compare Figs. 1, 4 and 6), pointing out a specific role of *AaCHS1a *in chitin synthesis for the serosal cuticle by serosal cells, as indicated. It is highly unlikely that *AaCHS1a *early expression, from 9 until 24 HAE, is related to larval cuticle chitin synthesis: 24-hour old embryos are at the germ band retraction stage (data not shown), and cuticle formation starts later. Raminani and Cupp [43] showed *Aedes aegypti *larval cuticle production starts at 47–52% of development. Accordingly, the later expression of both *AaCHS1a *and *AaCHS1b *at 31 HAE occurs at 52% of the total embryogenesis time period. This late expression of both *AaCHS1a *and *AaCHS1b*, but not the early *AaCHS1a *expression, would have a role in organogenesis and larval cuticle synthesis, as described for *D. melanogaster *[29,44,45]. In this later species, *Dm_kkv *(CHS-A gene) deletion is embryonic lethal [29,30], and the expression of *kkv *is concomitant with chitin production for the larval cuticle [29]. These data suggest a crucial role of CHS-A genes in Dipteran embryogenesis. In *D. melanogaster *the gene *kkv *is expected to participate only in chitin synthesis of the larval cuticle, since Cyclorrhapha flies do not have a serosa (see below), therefore lacking a serosal cuticle.

### Serosa and the serosal cuticle role in insects

In insects, immediately after blastoderm formation, cells of the developing egg follow distinct fates, originating the embryonic (germ) rudiment and the serosa anlage where the embryonic rudiment will form the embryo itself and the amnion [8,46-48]. The serosa originates from an anterior or anterodorsal portion of the egg [8,32,47,49]. Following gastrulation the serosa completely envelopes the embryo and the yolk [2,8,10,50,51]. Dipterans from Nematocera and Brachycera taxa also form separate amnion and serosa, with the exception of the Cyclorrhapha (Brachycera) flies, such as *Musca *(Muscidae, Calyptratae) and *Drosophila *(Drosophilidae, Acalyptratae), that do not form amnion and serosa, only a reminiscent amnioserosa [10,51,52]. It is argued that the serosa is important for developing insect embryos, protecting it against bacterial challenge [53] or insecticides [54]. The absence of a functional serosa results in a complete everted (inside out) embryo as observed with RNAi for Tc-Zen2 and Of-Zen in *T. castaneum *and *O. fasciatus*, respectively [48,55]. Putative roles of serosa cells on yolk digestion [9,56] or in embryonic excretion [9] were also suggested.

**Figure 7:**
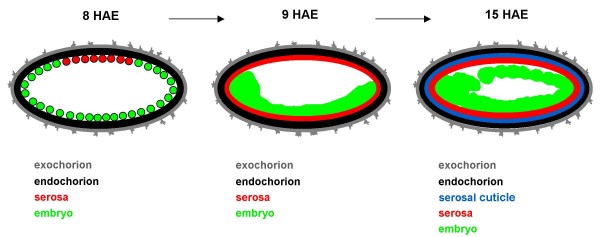
**Chronology of *Aedes aegypti *eggshell layers formation**. Schematic drawing of the structure of an embryo and egg covering (cross section). For the sake of simplicity the amnion is absent. Up to 8 HAE the embryo is surrounded by a composite external exochorion and an internal endochorion. At 9 HAE the cellular serosa develops around the embryo. Serosa secretes the serosal cuticle, which becomes localized between the serosa and the endochorion by 15 HAE.

In some mosquitoes, as in other insects (see below), after enclosing the entire embryo the serosa secretes the serosal cuticle (SC), a layer that is located above the serosa and below the endochorion [2,4], see Figure 7. In *Aedes *mosquitoes it has been previously shown that SC formation is related to egg desiccation resistance [2,4,5,7]. Orthoptera, Coleoptera and Lepidoptera insects also possess a SC [9,11,57], but its function in these insects is unknown. Some findings indicate that in the Coleoptera *Ocypus olens *the serosal cuticle may function as a water barrier for developing embryos [58]. Since the basal Hexapoda clade Archaeognatha also possesses a SC [46], it can be inferred that this is a primitive trait in insects. Taking this into account the absence of a serosa, and in consequence of a SC, would be a derived trait in Cyclorrhapha flies such as *D. melanogaster*.

Apart from its importance as the subject of study of the evolution of extra-embryonic membranes in insects [47], the serosa possesses the seminal role in *Ae. aegypti *of secreting the serosal cuticle. This layer is incriminated as important for desiccation resistance in Aedes embryos and probably has a similar role in other mosquito genera as well. Aedes eggs, when compared to other mosquito genera, can survive on dry for a much longer period of time [2]. We therefore speculate that SC contributes with different desiccation resistance levels in eggs from distinct Culicidae genera. We are currently working to address the existence and role of SC in other mosquitoes species.

### Serosal cuticle formation and physiological role in *Ae. aegypti *embryos

We have shown that the extra-embryonic serosa completely surrounds *Ae. aegypti *embryos at the beginning of germ band extension, at 9 HAE, followed by abrupt SC formation, between 11 and 13 HAE. This 2-hour interval corresponds to only 3.2% of total embryonic development.

The exposure of embryos to dry conditions for long periods before SC formation also results in complete blockage of hatching. By contrast, if the same procedure is performed after SC formation, all larvae are viable, even when embryos are left on dry from 20 to 140 HAE (i.e.: around 3 days after the end of embryogenesis, Fig. 2). Since after completion of embryogenesis the dormant larvae can resist many months on dry [3], it should be argued that the observed rate of survival after 3 days on dry indicates that after 20 HAE, a developing embryo is putatively able to survive also for many months on dry, as suggested before [1]. The data presented strongly argues in favor of an essential role of SC in protecting the developing embryo, and further the larvae, from desiccation. Irrespective of a direct relationship between SC formation and desiccation resistance acquisition, early embryos are not resistant to desiccation neither water impermeable, as recently claimed [17,18].

### On the waterproofing nature of desiccation resistance

It is important to underlie that neither the SC nor the synthesized chitin from SC may be solely responsible for impermeability, where the serosal cuticle-endochorion bonding would be the functional unity that confers desiccation resistance [4]. Additionally, it was claimed that a wax layer, as a part of the serosal cuticle, would be an important factor for desiccation resistance in *Ae. aegypti *eggs [1,2,4], in the same way wax is important for impermeability to the integument (exoskeleton) of insects [59,60]. Indeed, Slifer [11] observed two layers in the serosal cuticle of the grasshopper *Melanoplus differentialis*: the "yellow cuticle", a thin externalmost layer secreted first and a thicker layer, so-called "white cuticle" lying beneath. Chitin is present in the white cuticle and it was suggested that the yellow cuticle has wax in its composition [11,61,62]. In the Hemiptera *Rhodnius prolixus*, a wax layer is deposited around the embryo following serosa formation, insuring further resistance against desiccation [63].

We thus propose that the final, mature desiccation resistance will be achieved by the interaction of proteins from the endochorion with proteins, lipids and sugars present in the serosal cuticle. These interactions could be orchestrated by the process of sclerotization [64], which would be happening at the SC-endochorion bonding region. Obviously these interactions would occur after the SC was shed, and could be responsible for the observed differences in the kinetics of events related to desiccation resistance here described.

In the case of Culicidae embryonic development, the dynamics and the nature of the physiological processes that underlie eggshell impermeability acquisition are still poorly understood. Additional experiments will be needed to functionally confirm the putative SC role on desiccation resistance, the role of SC-endochorion interactions in the impermeable status of mosquito eggs as well as the presence of a SC wax layer in *Ae. aegypti*.

## Conclusion

Our findings indicate the serosal cuticle is associated to *Ae. aegypti *egg desiccation resistance acquisition, a phenomenon that abruptly develops between 11 and 13 hours after egglaying. It was also confirmed that, in addition to its known involvement in the peritrophic matrix and exoskeleton [59], chitin is present in the serosal cuticle, as early suggested [4,13]. *Aedes aegypti *has two Chitin Synthase genes, AaCHS1 and AaCHS2, only the former being expressed during embryogenesis. AaCHS1 has two mutually exclusive exons, *AaCHS1a *and *AaCHS1b*, both expressed at late embryogenesis, probably participating in organogenesis and embryo exoskeleton formation. However, only *AaCHS1a *is transcribed during SC development, thus providing chitin for the SC structural backbone, where SC on its turn is involved with desiccation resistance, an important factor for the adaptative success of *Ae. aegypti*, the dengue and urban yellow fever vector.

## Methods

### 1. Mosquitoes

*Aedes aegypti *mosquitoes from the Rockefeller strain, continuously reared in the laboratory at 26°C and 70% r.h., were adopted. Larvae received cat food (Friskies^®^, Purina, Camaquã, RS, Brazil), and adults were fed *ad libitum *with 10% sucrose solution. Blood feeding, necessary for egg production, was employed after female mosquitoes were sugar deprived for 24 hours, with anesthetized guinea pigs.

### 2. Synchronous egglaying

This method was adapted from Valencia et al [65]. Three to four days after blood-feeding, *Ae. aegypti *females were placed in a tube, anesthetized in ice for 1 minute and quickly transferred to a Whatman No. 1 paper disk, fit in the lid of a Petri dish (90 × 15 mm or 150 × 15 mm). The lid was then covered with the base of the dish, mosquitoes being allowed to recover for about 5–10 minutes. With the aid of the tip of a pipet introduced between the base and the lid, the filter paper was soaked with dechlorinated water, and the Petri dish was put in a humid chamber inside an incubator at 28°C protected from light. With this procedure egglaying started immediately. In all cases oviposition was permitted during 20-minutes, when Petri dishes were opened inside a large cage to release the mosquitoes. In some cases egglaying could be induced twice with the same females in the same oviposition cycle. Eggs were kept at 28°C until the required age, the onset being considered the end of the 20-minute egglaying period. Embryonic age was assigned as hours after egglaying (HAE). At 28°C *Ae. aegypti *embryogenesis is completed in 61.5 hours (Farnesi and Rezende, to be published elsewhere).

### 3. Analysis of desiccation resistance acquisition by air drying exposure

Eggs at different HAE were treated according to a protocol adapted from Valencia et al [65,66]: 50 eggs were aligned on a polycarbonate filter (2.5 cm diameter, 8 μm pore, Poretics Corporation), deposited above a drop of distilled water. The filter was then blotted on a Whatman No. 1 filter paper to remove all water, eggs were air-exposed during 15 minutes and the number of shrunken eggs was confirmed with a Zeiss stereomicroscope. The 15-minute air-drying was performed at 24–27°C with 40–45% relative humidity.

### 4. Analysis of desiccation resistance acquisition by viability of eggs left on dry at distinct embryonic ages

Synchronized developing eggs obtained as indicated in section 2 above were transferred from wet to dry conditions at different ages: 10, 15 and 20 HAE. In each case, groups of 40 eggs were kept on dry for varying periods: 25, 48 and 120 hours (see Fig. 2 for the assay design). Egg viability was then quantified through L1 larvae counting, after a hatching stimulus, performed according to Farnesi and Rezende (to be published elsewhere). In some experimental conditions the total test interval ("wet plus dry") remained below the presumptive embryogenesis completion period (61.5 hours). In these cases eggs were returned to a moist Whatman n°1 filter paper up to that period. For each experimental condition a parallel control sample was kept in moist filter paper up to 61.5 hours and then, if necessary, remained in the dry for a period of time equivalent to its experimental counterpart. The experiments were performed ate least in triplicates inside an incubator at 28°C with 40–80% relative humidity.

### 5. Sodium hypochlorite (NaOCl) digestion of egg chorion

Eggs aged 11 and 13 HAE were treated with 30% NaOCl (containing approximately 3.6% active chlorine at final concentration) for 15–30 minutes and observed under a Zeiss stereomicroscope.

### 6. Embryo morphology analysis

Synchronously laid eggs of different ages were fixed and clarified according to Trpis [67]. Embryos inside the resulting transparent eggshells were observed under an Axioskop 40 microscope (Zeiss) with phase contrast and a Stereo Discovery V.12 stereoscope (Zeiss). Embryonic stages were identified in compliance with previous authors [36,43,52,68,69].

Attempts to observe serosa nuclei with DAPI were unsuccessful due to embryo autofluorescence and incompatibility of the fixation protocol with DAPI staining.

### 7. Detection of chitin in the serosal cuticle

Calcofluor White M2R (Sigma, St. Louis, MO, also named Fluostain I or Fluorescent Brightener 28, Molecular Formula: C_40_H_44_N_12_O_10_S_2_, Catalog # F-3543), already used to identify chitin in yeast cells [70], nematode eggs [71] and *D. melanogaster *embryos [29], was available in our laboratory only in the acid form which is not directly soluble in water. An aqueous 1 mg/ml Calcofluor solution was prepared by the addition of some drops of 2 N KOH, resulting in a light yellow solution.

Embryos of 9 and 15 HAE were clarified and fixed as mentioned above. Serosal cuticle (SC) isolation from dormant, fully developed embryos was adapted from Harwood [13] and Judson and Hokama [6]. Complete digestion of exo and endochorion of fully developed eggs was attained with 30% commercial sodium hypochlorite (NaOCl) during 25–30 minutes inside a Falcon cell strainer (70 μm Nylon, Becton Dickinson). The remaining SCs, with larvae inside, were then thoroughly washed with water. This last procedure breaks the SC, often at the line of dehiscence [6]. It should be noted that larvae that leave the SC remain in the sample and serve as a positive control for subsequent Calcofluor labeling.

Embryos of 9 and 15 HAE together with isolated SCs were incubated with Calcofluor during 10 minutes in the dark, thoroughly washed with 25 mM pH 6.2 sodium phosphate buffer and analyzed under a Zeiss LSM 510 META Confocal laser scanning microscope with a violet diode excitation laser (405 nm) and a bandpass BP 420–480 nm emission filter.

Isolated SCs were also incubated with 5 μg/ml WGA-FITC (EY Laboratories) in a solution of PBS with 2% BSA (w/v) for 1 hour at room temperature. Serosal cuticles were then thoroughly washed in this solution, mounted and analyzed with dark field or fluorescence microscopy.

Chitin content of isolated SCs was determined as described elsewhere [39,72].

### 8. Cloning and sequencing of *Ae. aegypti *CHS1 gene fragments

We adopted the CHS nomenclature proposed by Merzendorfer [27], who renamed *Ae. aegypti *and *An. gambiae *genes: class A and class B genes are termed respectively CHS-1 and CHS-2, regardless of the gene discovery chronology. Accordingly, *Ae. aegypti *CHS described earlier by Ibrahim [41], is a class B gene, and will be referred to as AaCHS2.

PCR with degenerated primers (CSA3Fdeg: ACNAAYCCNTAYTGGAT and CSA3Rdeg: GGCCAYTTNACRTGDTA) was performed with *Ae. aegypti *genomic DNA to amplify CHS-A fragments homologous to the *D. melanogaster kkv *(GenBank Accession No. NM_079509) and the putative *An. gambiae AgCHS1 *(previously referred to as *AgCHS2*, GenBank Accession No. XM_321336). The amplified fragments were cloned and sequenced at Instituto Oswaldo Cruz on an ABI 377XL with a BigDye Terminator V3.0 (Applied Biosystems). Sequence analysis was conducted with GCG software (Wisconsin Package Version 10.0), NCBI website  and ClustalW.

### 9. AaCHS1 gene assembling and protein sequence analyses

Complete *AaCHS1 *DNA sequence information was initially obtained from a genomic Blast  using TBLASTN with the *kkv *(*D. melanogaster *CHS-A) sequence against *Ae. aegypti *genome. The genomic region of AaCHS1 was located inside a whole genome shotgun (WGS) sequence (GenBank accession No. AAGE02004132). A partial cds of AaCHS1 is deposited in GenBank (accession No. XM_001662150). This partial mRNA lacks the first exon plus the last putative 30 bases, before the stop codon of the deduced protein, as interpreted by the WGS sequence and alignment (see Fig. 4). Multiple alignments of deduced amino acid sequences were assembled using ClustalW software. The protein sequence was analyzed for transmembrane helices with the TMHMM v.2.0 software available at .

### 10. RNA expression analysis of AaCHS1 by qPCR

Total RNA was extracted with Trizol reagent (Invitrogen) according to manufacturer instructions and cDNA was synthesized with the TaqMan Reverse Transcriptase Kit (Applied Biosystems) with the Not I-d(T)_18 _primer.

Quantitative Real-time PCR (qPCR) was carried out in ABI Prism 7000 Sequence Detection Systems (Applied Biosystems) with the Power SYBR Green PCR Master Mix (Applied Biosystems).

The primers utilized for specific *AaCHS1 *expression (*AaCHS1a *or *AaCHS1b*, using Exon 6a or 6b, respectively, see Fig. 5b) were CS9ExA (5' CAAGGACAATATCCACGTCAAG 3') and CS9R (5' AGGCCAAGATATGCGACAG 3') for *AaCHS1a*, and CS10ExB (5' ACCTATGACGAAGTGACAGCC 3') and CS10R (5' TTGCAACCCCAGTTCAGCTC 3') for *AaCHS1b*. Primers 5aeexpRP and 3aeaquaRP1b [73] were employed to amplify the constitutive gene *rp49*, used as an internal normalizer for qPCR. 24 and 52 HAE values were designated as calibrators for the expression levels of AaCHS1a and AaCHS1b, respectively.

## Authors' contributions

GLR conceived, designed and carried out most experiments and drafted the manuscript. AJM conceived part of the study and designed and carried out the air-drying experiments. CG helped with RNA samples and to design the qPCR experiment. LCF helped with viability experiments and carried out the chitin quantitation on isolated serosal cuticles. MPM and AAP participated in the design and coordination of experiments and supervised GLR. DV is the principal investigator, conceived of the study and participated in the design and coordination of experiments, and helped to write the manuscript. All authors read, made comments and approved the final manuscript.

## Supplementary Material

Additional file 1 Expression of *AaCHS1 *and *AaCHS2 *during embryogenesis). Click here for file

Additional file 2 Alignment of predicted protein sequences of Dipteran CHS1 sequence. Click here for file

Additional file 3 Embryo morphology at late embryogenesis. Click here for file
