# Supplementary material for: Embryonic desiccation resistance in Aedes aegypti: presumptive role of the chitinized Serosal Cuticle
Source: BMC Dev Biol. 2008 Sep 13;8:82. doi: 10.1186/1471-213X-8-82 (PMC2561029; doi:10.1186/1471-213X-8-82)
Supplement: Additional file 1 — Expression of AaCHS1 and AaCHS2 during embryogenesis). [file 1471-213X-8-82-S1.pdf]

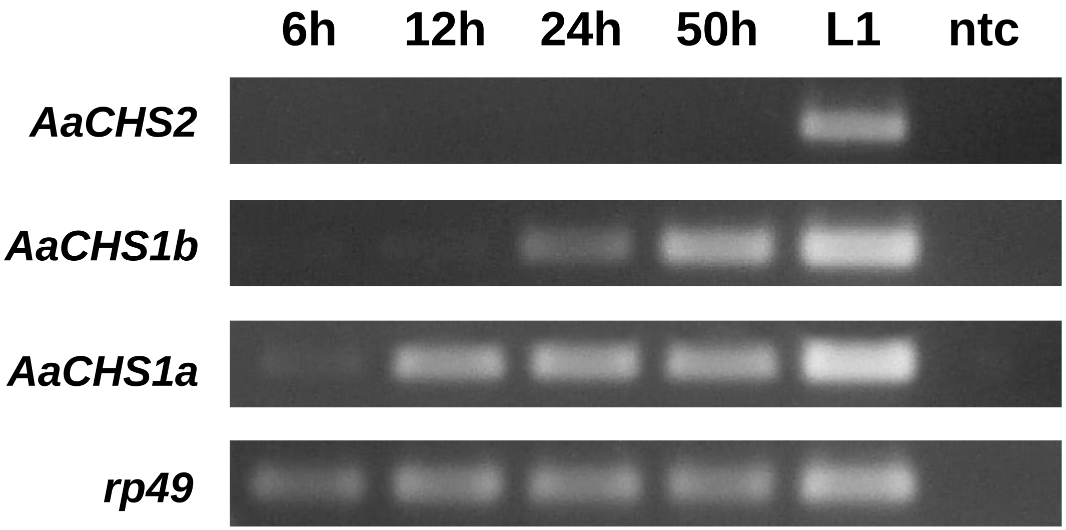

Additional Figure 1: Expression of *AaCHS1* and *AaCHS2* during embryogenesis. The constitutive gene rp49 is used as a control. *AaCHS1* has two splice variants, *AaCHS1a* and *AaCHS1b* (see main text). 6h - 50h: hours of embryonic development. L1: feeding stage of L1 larvae. ntc: non template control. Primers used to amplify AaCHS2 were CS2AF (5' AAGAACGACACGATAAAGCC 3') and CS2AR (5' TTGTTGATGAGCGTCTTCAC 3')
