# Supplementary material for: Embryonic desiccation resistance in Aedes aegypti: presumptive role of the chitinized Serosal Cuticle
Source: BMC Dev Biol. 2008 Sep 13;8:82. doi: 10.1186/1471-213X-8-82 (PMC2561029; doi:10.1186/1471-213X-8-82)
Supplement: Additional file 2 — Alignment of predicted protein sequences of Dipteran CHS1 sequence. [file 1471-213X-8-82-S2.pdf]

```
                  1                                               ▼        47
AaCHS1            MSAIRHRPLATQ-------------NESDDNFTDDESTPLTQDIYGGSQRTVQETKGWDV
AgCHS1            MSAIRHRPLANGP------------NESDDNFSDDESTPLTHDIYGGSQRTVQETKGWDV
Dm_kkv            MSAMRHRPMAPPGQGPGAGTAGEHVDSDDNNFTDDESSPLTHDIYGGSQRTIQETKGWDV
                  ***:****:*               :..*:**:****:***:*********:********

                  48                                                        107
AaCHS1            FRDPPIKEDTGSMADQACLELTIKILKIFAYLLTFVIVLTGGVVAKGCVLFMSSQLKRDR
AgCHS1            FRDPPIKEDTGSMADQACLDLTIKILKIFAYLITFVIVLLGGVVAKGCVLFMSSQLRRDR
Dm_kkv            FRDPPIKIETGSTANQECLELTVKILKIFAYVITFIIVLTGGVIAKGTMLFMTSQVRKDK
                  ******* :*** *:* **:**:*******::**:*** ***:*** :***:**:::*:

                  108                                                       167
AaCHS1            KITYCNRDLARDKQFVATLPEEERIAWMWALMIAFAVPEIGTFIRSTRICFFKSMKKPFK
AgCHS1            KITYCNRDLARDKSFIVSLPEEERIAWMWALMIAFAVPEIGTFIRSTRICFFKSMKKPLK
Dm_kkv            KMEYCNKDLGRDKSFVVRLPEEERVAWIWALLIAYALPEIGALIRSARICFFKTFKVPKT
                  *: ***:**.***.*:. ******:**:***:**:*:****::***:******::* * .

                  168                                                       227
AaCHS1            SHFLLVFLMESFHTIGLVLLFFVVLPEVDSVKGAMLTNCLCVIPGLLGMFSRTNKEGKRA
AgCHS1            SHFLLVFLMESFHTIGLVLLFFVVLPEVDSVKGAMLTNCLCVIPGMLGLFSRTNKEGKRA
Dm_kkv            GHFLFVWLMESLSAVGMALLMFVVLPQIDAIQGAMLTNCLCVVPGIFGLLSRTSKEGKRF
                  .***:*:****: ::*:.**:*****::*:::**********:**::*::***.*****

                  228                                                       287
AaCHS1            VKSIVDLAAIAAQITGFVVWPLLENRPVLWLIPVSALLTSCGWWENYVSPQSPFGFIRAM
AgCHS1            VKSIVDLAAIAAQITGFIVWPLLENRPVLWLIPVSALLTSCGWWENYVSPQSPFSFVRSL
Dm_kkv            VKVIIDLAAVAAQVTGLVIWPLLENRRELWVIPVACVMISCGWWENYVSPQSPLGLVRAL
                  ** *:****:***:**:::*******  **:***:.:: *************:.::*::

                  288                                                       347
AaCHS1            GRVKEDLKQTRYFTYMFLSLWKVMLFFCIVLVIMFFNGDEVANLFSLFGVGFGPHKIVVE
AgCHS1            GRVKEDLKQTRYFTYMFLSVWKILLLFCFVSVILFVRGDEVANLFSLFGAGYGPHKIVVE
Dm_kkv            GRIKEEMKYTRYFCHIFLSIWKILLFFTVTLLIYWAQGEEPGNLFAMYGDAFGPHKIIVY
                  **:**::* **** ::***:**::*:* .. :* : .*:* .***:::* .:*****:*

                  348                                                       407
AaCHS1            EVATAFSSALPDLVEASQAGDTVDIDASYNTVTYVLIIQILGAYLCYVFGKFACKILIQG
AgCHS1            EVALPFSSALPDLVEAAQAVDTIDIDAAYNTVTYVLIIQILAAYLCYIFGKFACKILIQG
Dm_kkv            ELPAGLGGVLPDTLESAN-IDTVDVDAAYNTVVYVLLLQIFGAYLCYIFGKFACKILIQG
                  *:.  :...*** :*:::  **:*:**:****.***::**:.*****:************

                  408                                                       467
AaCHS1            FSYAFPVNLTIPVAISLLIAACGIRNDDPCFFHGSIPDYLFFESPPVFRLSDFASRQMAW
AgCHS1            FSYAFPVNLTVPVAISLLIAACGIRNDDPCFFHGSIPDYLFFESPPVFRLNDFASRQMAW
Dm_kkv            FSYAFPVSLTVPLSVTFLIAACGIRIDDPCFFHDTIPDYLFFTSPSNFRFNNFVTEQMAW
                  *******.**:*::::******** *******.:******* **. **:.:*.:.****

                  468                                                       527
AaCHS1            AWLLWLLSQTWITLHIWTPKCERLANTEKLFVTPMYNALLIDQSMALNRRRDDQADVKTE
AgCHS1            AWLLWLLSQTWITLHIWTPKCERLANTEKLFVTPMYSALLIDQSMAMNRRRDDQADVKTE
Dm_kkv            AWILWLLSQTWIALHIWTPKCERLATTEKLFVQPMYSSLLIDQSMALNRRRDDQADVKTE
                  **:*********:***********.****** ***.:*********:************

                  528                                                       586
AaCHS1            DLAEIEKEKGDEYYETISVHTDGSALP-RPSVKSSDHITRIYACATLWHETKEEMIVFLK
AgCHS1            DLAEIEKEKGDEYYETISVHTDGSALP-RPSVKSSDHITRIYACATLWHETKEEMMVFLK
Dm_kkv            DLSEIEKEKGDEYYETISVHTDRSSAPNKPSIKSSDNITRIYSCATMWHETKDEMIEFLK
                  **:****************** *: * :**:****:*****:***:*****:**: ***
```

```
                587                                                        646
AaCHS1          SIMRMDEDQCARRVAQKYLRIVDPDYYEFESHIFFDDAFEISDHSDDDIQCNRFVKILID
AgCHS1          SIMRMDEDQCARRVAQKYLRIVDPDYYEFETHIFFDDAFEISDHSDEDIQCNRFVKILVD
Dm_kkv          SIMRMDEDQCARRVAQKYLRVLDPDYYEFETHIFFDDAFEISDHSDDDIQCNRFVKLLIA
                *******************::********:**************:*********:*:

                647                                                        706
AaCHS1          TIDEAASEVHQTNIRLRPPKKYPTPYGGRLVWTLPGKTKLIAHLKDKDRIRHRKRWSQVM
AgCHS1          TIDEAASEVHQTNIRLRPPKKYPTPYGGRLVWTLPGKTKMIAHLKDKDRIRHRKRWSQVM
Dm_kkv          TMDEAASEIHQTTIRLRPPKKYPTPYGGRLVWTLPGKTKFITHLKDKDRIRHRKRWSQVM
                *:******:***.************************:*:*********************

                707                                                        766
AaCHS1          YMYYLLGHRLMELPISVDRKDVMAENTYLLTLDGDIDFNPSAVTLLVDLMKKNKNLGAAC
AgCHS1          YMYYLLGHRLMELPISVDRKEVMAENTYLLTLDGDIDFNPSAVTLLIDLMKKNKNLGAAC
Dm_kkv          YMYYLLGHRLMELPISVDRKDAIAENTYLLTLDGDIDFKPNAVTLLVDLMKKNKNLGAAC
                *******************:.:**************:*.*****:*************

                767                                                        826
AaCHS1          GRIHPIGSGPMVWYQKFEYAIGHWLQKATEHMIGCVLCSPGCFSLFRGKGLMDDNVMRKY
AgCHS1          GRIHPIGSGPMVWYQKFEYAIGHWLQKATEHMIGCVLCSPGCFSLFRGKGLMDDNVMRKY
Dm_kkv          GRIHPVGSGPMVWYQLFEYAIGHWLQKATEHMIGCVLCSPGCFSLFRGKALMDDNVMKKY
                *****:********* *******************************.*******:**

                827                                                        886
AaCHS1          TTRSDEARHYVQYDQGEDRWLCTLLLQRGYRVEYSAASDAYTHCPEGFNEFYNQRRRWVP
AgCHS1          TTRSDEARHYVQYDQGEDRWLCTLLLQRGYRVEYSAASDAYTHCPEGFNEFYNQRRRWVP
Dm_kkv          TTRSDEARHYVQYDQGEDRWLCTLLLQRGYRVEYSAASDAYTHCPEGFNEFYNQRRRWVP
                ************************************************************

                887                                                        946
AaCHS1          STIANIMDLLMDYKRTIKINDNISLLYIFYQMMLMGGTILGPGTIFLMLVGAFVAAFKID
AgCHS1          STIANIMDLLMDYRRTIKINDNISLLYIFYQMMLMGGTILGPGTIFLMLVGAFVAAFKID
Dm_kkv          STIANIMDLLADAKRTIKINDNISLLYIFYQMMLMGGTILGPGTIFLMLVGAFVAAFRID
                ********** * :*****************************************:**

                947                                                        1006
AaCHS1          NWTSFYYNIIPILLFMLVCFTCKSNIQLLLAQILSTVYALIMMAVIVGTALQLGEDGIGS
AgCHS1          NWTSFYYNIIPIMLFMLVCFTCKSNIQLLVAQILSTVYALIMMAVIVGTALQLGEDGIGS
Dm_kkv          NWTSFHYNIVPILAFMFICFTCKSNIQLFVAQVLSTAYALIMMAVIVGTALQLGEDGIGS
                *****:***:**: **::**********::**:***.*********************

                1007                                                       1066
AaCHS1          PSAIFLIAMTGSFFIAACLHPQEFWCIASGLIYLLSIPSMYLLLILYSIINLNVVSWGTR
AgCHS1          PSAIFLIAMTGSFFIAACLHPQEFWCIASGIIYLLSIPSMYLLLILYSIINLNVVSWGTR
Dm_kkv          PSAIFLISMVGSFFIAACLHPQEFWCITCGLIYLLSIPSMYLLLILYSIINLNVVSWGTR
                *******:*.****************:.*:****************************

                1067                                                       1126
AaCHS1          EVVAKKTKKELEQEKKEAEEAAKRVKQKSLLGFLQGGAGSNADEEGSIEVSIAGLFRCLL
AgCHS1          EVVAKKTKKEMEQEKKDAEEAAKRAKQKSLLGFLQGGVGNGSDEEGSIDISIAGLFRCLL
Dm_kkv          EVVAKKTKKELEAEKKAAEEAKKRVKQKSMLSFLQSGIGDNGDEEGSVEFSLAGLFRCIF
                **********:* *** **** **.****:*.***.* *...*****::.*:******::

                1127                                                       1182
AaCHS1          CTHGKTTDEKAQLVHIGDALDSITKKIENLEKHIDPHGHHT----RKRTASAGSRDHHLG
AgCHS1          CTHGKTTDEKAQLIHIADALDAITKKIENLEKHIDPHGHHT----RKRTASAGSKDHHLG
Dm_kkv          CTHGKTSDEKQQLTSIAESLDTIKHRMDTIESAVDPHGHHASRHGRRRTTSSGSKDHHLL
                ******:*** **  *.::**:*.::::.:*. :******:    *:**:*:**:****
```

```
                  1183                                                  1241
AaCHS1          -SVAEDSEEEDEQSDSETSTLQRNERDFLTNPYWIEDPDLKKGEVDFISSTEIQFWKDLI
AgCHS1          -SVAEDTEDDDEDEDSETSTLQRDERDFLTNPYWIEDPDLKKGEVDFISSTEIQFWKDLI
Dm_kkv          TSVAEKSGDESDESDSDTSAEPKQERDFLTNPYWIEDPDVRKGEVDFLSSTEIQFWKDLI
                 ****.: ::.::.**:**:  ::***************::******:************

                1242                                                   1302
AaCHS1          DQYLYPIDQNKEEQARIAHDLKELRDSSVFGFIMINALFVLIVFLLQLNKDNIHVKWPLG
AgCHS1          DKYLYPIDQNKEEQARIAVDLKELRNKSVFAFFMFNALFVLIVFLLQLNKDKLHIIWPLG
Dm_kkv          DQYLYPIDNDPVEQARIAKDLKELRDSSVFAFFMINALFVLIVFLLQLNKDNIHVKWPFG
                *:******::  ****** ******:.***.*:*:****************::*: **:*

                1303                                                   1362
AaCHS1          VKTNITYDEATQEVHISKEYLQLEPIGLVFVFFFALILIIQFTAMLFHRFGTLSHILAST
AgCHS1          VKTNITYDEVTAEVHISKEYLQLEPIGLVFVFFFALILIIQFVAMMFHRFGTLSHILAST
Dm_kkv          VRTNITYDESTQEVHISKEYLQLEPIGLVFVFFFALILIIQFTAMLFHRFGTISHILAST
                *:******* * ******************************.**:******:*******

                1363                                                   1422
AaCHS1          ELNWGCNKKPEELSQDALIDKHAVEIVKNLQRLQGIDGDYDNDSGSGPDRIARRRTIQNL
AgCHS1          ELNWACNKKPEELSQDALIDKHAVEIVKNLQRLQGIDGDYDNDSGSGPDRIARRRTIQNL
Dm_kkv          ELNF-CKKKSEDLTQDQLIDKHAVEIVKNLQRLQGIDGDYDNDSGSGPDRIARRKTIQNL
                ***: *:**.*:*:** *************************************:*****

                1423                                                   1481
AaCHS1          EKARQPRRQIGTLDVAFKKRFLKLTAD-ENNAGTPILTRRMTMRRETIRALEVRKNSVMA
AgCHS1          EKARQPRRQIGTLDVAFKKRFLKLTAD-ENNTATPILTRRMTMRRETIRALEVRKNSVMA
Dm_kkv          EKARQPRRQIGTLDVAFKKRFLKLTADAENNPATPILTRRLTMRAETIRALEVRKNSVMA
                *************************** ***..*******:*** ***************

                1482                                                   1538
AaCHS1          ERRKSQMQTLGANNEYGIT-GVNNTNNNAPQ--RPLRTSNAGVSVKDIFNVNGGPGGDIY
AgCHS1          ERRKSQMQTLGANNEYGIT-GVPNGNNNAPP--RPTRTSNAGVSVKDIFNVNGGPGGEIY
Dm_kkv          ERRKSAMQTLGAKNEYGITTGAPINNNGALPNQRSGRVSNAGISIKDVFNVNGGGAEQIY
                ***** ******:****** *.   **.*    *. *.****:*:**:****** . :**

                1539                                          1576
AaCHS1          GVTG--QVNQAYEPVIEDD-DRNSLRLQPRN-------QVTWSNS-NGRL-------
AgCHS1          GVTG--QVNQAYEPVIEDD-DRNSLRLQPRN-------QVTWGNNGNARL-------
Dm_kkv          GSNGGGTINQGYEHVIDEDGDGNSLRLTTRNPHPHPHHQVSWGQNTNGGGGNGTGRL
                * .*   :**.** **::* * ***** .**       **:*.:. *.
```

Additional Figure 2: Alignment of predicted protein sequences of Dipteran CHS1 sequence. Alignment of predicted Chitin Synthase 1 (CHS-A) amino acid sequence of *Ae. aegypti* (AaCHS1), *An. gambiae* (AgCHS1) and *D. melanogaster kkv* using ClustalW software. ▼ indicates the position of the first intron (28,587 bp) in the *AaCHS1* coding sequence. The region highlighted in gray corresponds to AaCHS1 exon 6a (see Fig. 5). Symbols below the aligned amino acid sequences indicate identical (*), highly conserved (:), and conserved (.) residues.
