# Supplementary material for: Embryonic desiccation resistance in Aedes aegypti: presumptive role of the chitinized Serosal Cuticle
Source: BMC Dev Biol. 2008 Sep 13;8:82. doi: 10.1186/1471-213X-8-82 (PMC2561029; doi:10.1186/1471-213X-8-82)
Supplement: Additional file 3 — Embryo morphology at late embryogenesis. [file 1471-213X-8-82-S3.pdf]

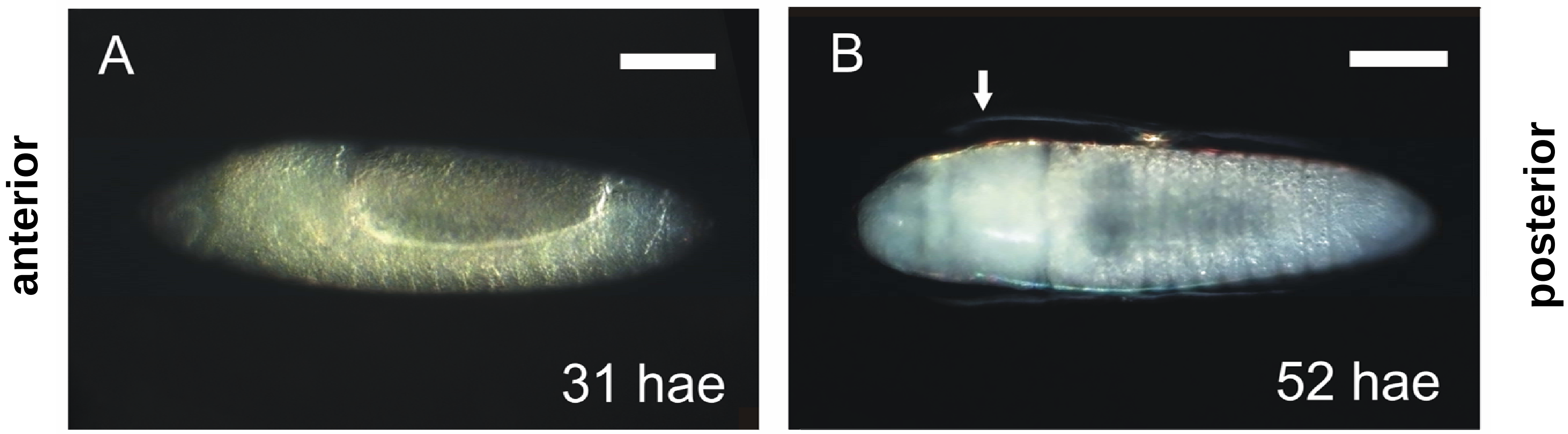

Additional Figure 3: Embryo morphology at late embryogenesis. (**A**) 31-HAE embryo at dorsal closure, lateral view. The plane of focus is inside the embryo, and thus the endochorion is not visible (**B**) 52-HAE embryo showing head, thoracic and abdominal segments, dorsal view. White arrow: endochorion. Bar = 100 µm
